# Methyl 4-hydr­oxy-2*H*-1,2-benzothia­zine-3-carboxyl­ate 1,1-dioxide

**DOI:** 10.1107/S1600536808028584

**Published:** 2008-09-13

**Authors:** Waseeq Ahmad Siddiqui, Saeed Ahmad, Hamid Latif Siddiqui, Mujahid Hussain Bukhari, Masood Parvez

**Affiliations:** a Department of Chemistry, University of Sargodha, Sargodha, Pakistan; b Department of Chemistry, University of Science and Technology, Bannu, Pakistan; c Institute of Chemistry, University of the Punjab, Lahore, Pakistan; d Department of Chemistry, The University of Calgary, 2500 University Drive NW, Calgary, Alberta, Canada, T2N 1N4

## Abstract

The asymmetric unit of the title compound, C_10_H_9_NO_5_S, contains two independent mol­ecules. The heterocyclic thia­zine rings in both mol­ecules adopt half-chair conformations, with the S atoms in each mol­ecule displaced by 0.455 (3) and 0.539 (3) Å and the N atoms displaced in the opposite direction by 0.214 (3) and 0.203 (3) Å, from the planes defined by the remaining ring atoms. The crystal structure is stabilized by O—H⋯O, N—H⋯O and C—H⋯O hydrogen bonds involving both inter- and intra­molecular inter­actions.

## Related literature

For related literature, see: Banerjee & Sarkar (2002 ▶); Cremer & Pople, 1975 ▶; Hirai *et al.* (1997 ▶); Khalil *et al.* (2000 ▶); Myung *et al.* (2002 ▶); Siddiqui *et al.* (2006 ▶, 2008 ▶).
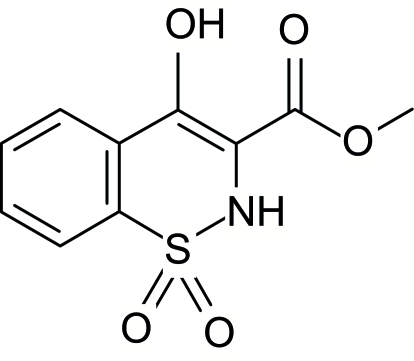


## Experimental

### 

#### Crystal data

C_10_H_9_NO_5_S
*M* *_r_* = 255.24
Triclinic, 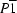

*a* = 7.777 (2) Å
*b* = 10.932 (4) Å
*c* = 12.890 (4) Å
α = 105.569 (16)°
β = 94.588 (15)°
γ = 97.763 (16)°
*V* = 1038.2 (6) Å^3^
*Z* = 4
Mo *K*α radiation
μ = 0.32 mm^−1^
*T* = 173 (2) K
0.24 × 0.22 × 0.16 mm

#### Data collection

Nonius KappaCCD diffractometer
Absorption correction: multi-scan (*SORTAV*; Blessing, 1997 ▶) *T*_min_ = 0.927, *T*_max_ = 0.950
8716 measured reflections
4693 independent reflections
4191 reflections with (*I*) > 2.0 σ(*I*)
*R*_int_ = 0.019

#### Refinement

*R*[*F* ^2^ > 2σ(*F* ^2^)] = 0.033
*wR*(*F* ^2^) = 0.088
*S* = 1.03
4693 reflections
321 parameters
H atoms treated by a mixture of independent and constrained refinement
Δρ_max_ = 0.39 e Å^−3^
Δρ_min_ = −0.41 e Å^−3^

### 

Data collection: *COLLECT* (Hooft, 1998 ▶); cell refinement: *HKL* *DENZO* (Otwinowski & Minor, 1997 ▶); data reduction: *SCALEPACK* (Otwinowski & Minor, 1997 ▶); program(s) used to solve structure: *SHELXS97* (Sheldrick, 2008 ▶); program(s) used to refine structure: *SHELXL97* (Sheldrick, 2008 ▶); molecular graphics: *ORTEP-3 for Windows* (Farrugia, 1997 ▶); software used to prepare material for publication: *SHELXL97*.

## Supplementary Material

Crystal structure: contains datablocks Global, I. DOI: 10.1107/S1600536808028584/lh2686sup1.cif

Structure factors: contains datablocks I. DOI: 10.1107/S1600536808028584/lh2686Isup2.hkl

Additional supplementary materials:  crystallographic information; 3D view; checkCIF report

## Figures and Tables

**Table 1:** Hydrogen-bond geometry (Å, °)

*D*—H⋯*A*	*D*—H	H⋯*A*	*D*⋯*A*	*D*—H⋯*A*
O1—H1*O*⋯O4	0.81 (2)	1.86 (2)	2.600 (2)	152 (2)
N1—H1*N*⋯O9	0.81 (2)	2.22 (2)	2.994 (2)	162 (2)
O6—H6*O*⋯O9	0.81 (2)	1.91 (2)	2.634 (2)	147 (2)
N2—H2*N*⋯O3^i^	0.83 (2)	2.13 (2)	2.966 (2)	175 (2)
C4—H4⋯O8^ii^	0.95	2.36	3.259 (2)	158
C20—H20*A*⋯O2^i^	0.98	2.51	3.267 (2)	134

Symmetry codes: (i) 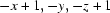; (ii) 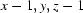.

**Fig. 1.**
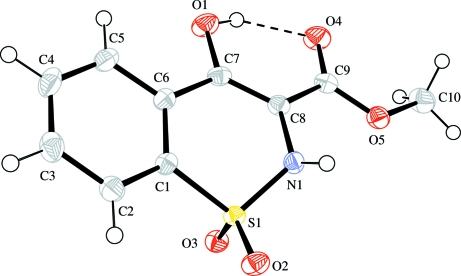
ORTEP-3 (Farrugia, 1997) drawing of molecule a with displacement ellipsoids plotted at 50% probability level.

**Fig. 2.**
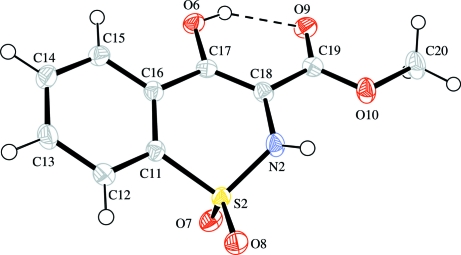
ORTEP-3 (Farrugia, 1997) drawing of molecule b with displacement ellipsoids plotted at 50% probability level.

**Fig. 3.**
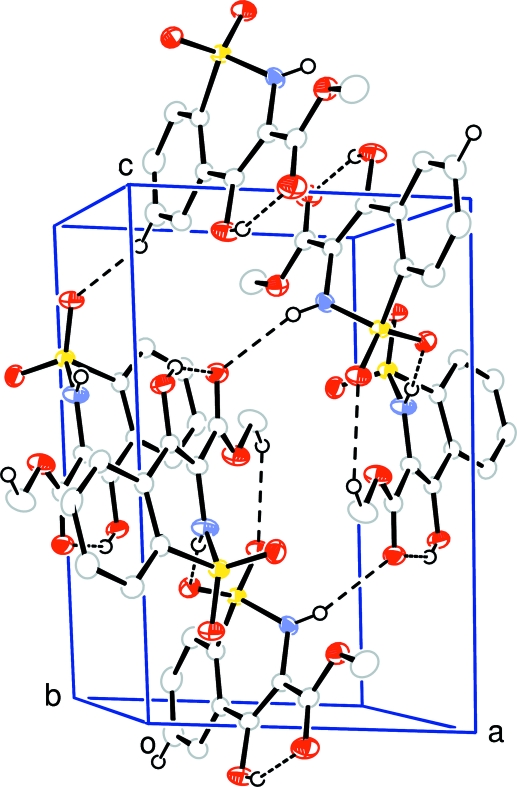
Part of the crystal structure showing H-bonding interactions (classical in red, non-classical in green and intramolecular in black) indicated by dashed lines, H-atoms not involved in H-bonds have been excluded.

## supplementary crystallographic information

### Comment

The 1,2-benzothiazine-3-carboxamide 1,1-dioxide derivatives belong to oxicams, a new class of non-steroidal anti-inflammatory drugs (NSAIDs). They are important for their analgesic and anti-inflammatory activities (Hirai *et al.*, 1997, Khalil *et al.*, 2000; Myung *et al.*, 2002). Besides great therapeutic potential, these are very motivating polyfunctional heterocyclic molecules by virtue of their dynamic structural features, which include different tautomeric forms and their possible polymorphism (Banerjee *et al.*, 2002). Continuing our investigations in this important field, (Siddiqui *et al.*, 2006, 2008), we now report the crystal structure of the title compound, (I), in this paper.

An asymmetric unit of (I) contains two independent molecules presented in Figures 1 (molecule a) and 2 (molecule b). The heterocyclic thiazine rings in both molecules adopt half-chair conformations, with atoms S1 and N1 in molecule a and atoms S2 and N2 in molecule b displaced by -0.455 (3), 0.214 (3), -0.539 (3) and 0.203 (3) Å, from the planes defined by C1/C6/C7/C8 and C11/C16/C17/C18, respectively; the puckering parameters (Cremer & Pople, 1975) are *Q* = 0.4365 (12) and 0.4901 (12) Å, θ = 61.8 (2) and 64.1 (2)° and φ = 19.6 (2) and 17.5 (2)°, respectively. Similar conformations of the corresponding rings have been reported in some closely related compounds (Siddiqui *et al.*, 2008).

The structure is stabilized by classical as well as non-classical hydrogen bonding (Fig. 3). Details of the hydrogen bonding geometry have been provided in Table 1.

### Experimental

The synthesis of the title compound as an important intermediate in the synthesis of oxicams has been reported (Siddiqui *et al.*, 2006). Crystals suitable for crystallographic studies were obtained from a solution of MeOH by slow evaporation at 313 K.

### Refinement

Though all the H atoms could be distinguished in the difference Fourier map the H-atoms bonded to C-atoms were included at geometrically idealized positions and refined in riding-model approximation with the following constraints: aryl and methyl C—H distances were set to 0.95 and 0.98 Å, respectively, and *U*_iso_(H) = 1.2 *U*_eq_(C). The H-atoms bonded to N and O-atoms were allowed to refine with *U*_iso_(H) = 1.2 *U*_eq_(N/O). The final difference map was free of any chemically significant features.

### Crystal data

**Table:**

C_10_H_9_NO_5_S	*Z* = 4
*M**_r_* = 255.24	*F*(000) = 528
Triclinic, *P*1	*D*_x_ = 1.633 Mg m^−^^3^
Hall symbol: -P 1	Mo *K*α radiation, λ = 0.71073 Å
*a* = 7.777 (2) Å	Cell parameters from 8716 reflections
*b* = 10.932 (4) Å	θ = 3.3–27.5°
*c* = 12.890 (4) Å	µ = 0.32 mm^−^^1^
α = 105.569 (16)°	*T* = 173 K
β = 94.588 (15)°	Block, colorless
γ = 97.763 (16)°	0.24 × 0.22 × 0.16 mm
*V* = 1038.2 (6) Å^3^	

### Data collection

**Table:**

Nonius KappaCCD diffractometer	4693 independent reflections
Radiation source: fine-focus sealed tube	4191 reflections with (*I*) > 2.0 σ(*I*)
graphite	*R*_int_ = 0.019
ω and φ scans	θ_max_ = 27.5°, θ_min_ = 3.3°
Absorption correction: multi-scan (SORTAV; Blessing, 1997)	*h* = −10→10
*T*_min_ = 0.927, *T*_max_ = 0.950	*k* = −14→13
8716 measured reflections	*l* = −16→16

### Refinement

**Table:**

Refinement on *F*^2^	Primary atom site location: structure-invariant direct methods
Least-squares matrix: full	Secondary atom site location: difference Fourier map
*R*[*F*^2^ > 2σ(*F*^2^)] = 0.033	Hydrogen site location: inferred from neighbouring sites
*wR*(*F*^2^) = 0.088	H atoms treated by a mixture of independent and constrained refinement
*S* = 1.03	*w* = 1/[σ^2^(*F*_o_^2^) + (0.041*P*)^2^ + 0.6*P*] where *P* = (*F*_o_^2^ + 2*F*_c_^2^)/3
4693 reflections	(Δ/σ)_max_ = 0.001
321 parameters	Δρ_max_ = 0.39 e Å^−^^3^
0 restraints	Δρ_min_ = −0.41 e Å^−^^3^

### Special details

**Table:**

Geometry. All e.s.d.'s (except the e.s.d. in the dihedral angle between two l.s. planes) are estimated using the full covariance matrix. The cell e.s.d.'s are taken into account individually in the estimation of e.s.d.'s in distances, angles and torsion angles; correlations between e.s.d.'s in cell parameters are only used when they are defined by crystal symmetry. An approximate (isotropic) treatment of cell e.s.d.'s is used for estimating e.s.d.'s involving l.s. planes.
Refinement. Refinement of *F*^2^ against ALL reflections. The weighted *R*-factor *wR* and goodness of fit *S* are based on *F*^2^, conventional *R*-factors *R* are based on *F*, with *F* set to zero for negative *F*^2^. The threshold expression of *F*^2^ > σ(*F*^2^) is used only for calculating *R*-factors(gt) *etc*. and is not relevant to the choice of reflections for refinement. *R*-factors based on *F*^2^ are statistically about twice as large as those based on *F*, and *R*- factors based on ALL data will be even larger.

### Fractional atomic coordinates and isotropic or equivalent isotropic displacement parameters (Å^2^)

**Table:**

	*x*	*y*	*z*	*U*_iso_*/*U*_eq_	
S1	0.30114 (5)	0.07908 (3)	0.24748 (3)	0.01734 (10)	
O1	0.23427 (16)	−0.14432 (12)	−0.08819 (9)	0.0293 (3)	
H1O	0.263 (3)	−0.213 (2)	−0.0882 (18)	0.035*	
O2	0.40121 (15)	0.17533 (10)	0.33806 (9)	0.0245 (2)	
O3	0.14058 (14)	0.00794 (10)	0.26334 (9)	0.0218 (2)	
O4	0.37308 (17)	−0.31701 (11)	−0.02096 (9)	0.0311 (3)	
O5	0.49305 (15)	−0.26084 (10)	0.15392 (9)	0.0243 (2)	
N1	0.42676 (17)	−0.02186 (12)	0.19684 (10)	0.0200 (3)	
H1N	0.528 (3)	−0.0058 (18)	0.2220 (16)	0.024*	
C1	0.25147 (19)	0.14330 (14)	0.13951 (12)	0.0192 (3)	
C2	0.2150 (2)	0.26789 (15)	0.16002 (13)	0.0247 (3)	
H2	0.2298	0.3231	0.2320	0.030*	
C3	0.1567 (2)	0.31014 (17)	0.07309 (15)	0.0297 (4)	
H3	0.1312	0.3951	0.0855	0.036*	
C4	0.1354 (2)	0.22878 (18)	−0.03175 (14)	0.0303 (4)	
H4	0.0919	0.2579	−0.0903	0.036*	
C5	0.1764 (2)	0.10609 (17)	−0.05230 (13)	0.0251 (3)	
H5	0.1628	0.0520	−0.1247	0.030*	
C6	0.23809 (18)	0.06130 (15)	0.03369 (12)	0.0195 (3)	
C7	0.28587 (19)	−0.06738 (15)	0.01323 (12)	0.0207 (3)	
C8	0.37515 (19)	−0.10618 (14)	0.09083 (12)	0.0194 (3)	
C9	0.4123 (2)	−0.23777 (15)	0.06838 (12)	0.0220 (3)	
C10	0.5260 (3)	−0.39148 (16)	0.13991 (15)	0.0326 (4)	
H10A	0.5654	−0.4022	0.2106	0.039*	
H10B	0.6167	−0.4083	0.0917	0.039*	
H10C	0.4183	−0.4522	0.1079	0.039*	
S2	0.86977 (5)	0.34163 (3)	0.69700 (3)	0.01856 (10)	
O6	0.98831 (15)	0.24939 (12)	0.36870 (9)	0.0243 (2)	
H6O	0.932 (3)	0.180 (2)	0.3347 (17)	0.029*	
O7	0.72248 (14)	0.39245 (11)	0.66203 (9)	0.0253 (2)	
O8	0.89790 (15)	0.34412 (11)	0.80904 (9)	0.0252 (2)	
O9	0.77047 (14)	0.03249 (11)	0.33635 (9)	0.0261 (2)	
O10	0.70377 (16)	−0.02541 (11)	0.48543 (9)	0.0271 (3)	
N2	0.86481 (19)	0.19551 (13)	0.62361 (10)	0.0237 (3)	
H2N	0.869 (3)	0.138 (2)	0.6548 (16)	0.028*	
C11	1.05550 (19)	0.41894 (14)	0.65790 (12)	0.0187 (3)	
C12	1.1639 (2)	0.52218 (15)	0.73162 (13)	0.0235 (3)	
H12	1.1443	0.5475	0.8056	0.028*	
C13	1.3017 (2)	0.58800 (15)	0.69539 (14)	0.0268 (3)	
H13	1.3775	0.6586	0.7448	0.032*	
C14	1.3285 (2)	0.55026 (16)	0.58671 (14)	0.0263 (3)	
H14	1.4209	0.5970	0.5621	0.032*	
C15	1.2225 (2)	0.44570 (15)	0.51417 (13)	0.0226 (3)	
H15	1.2436	0.4204	0.4404	0.027*	
C16	1.08448 (19)	0.37690 (14)	0.54858 (12)	0.0188 (3)	
C17	0.97645 (19)	0.26167 (14)	0.47423 (12)	0.0189 (3)	
C18	0.8758 (2)	0.17361 (14)	0.51071 (12)	0.0202 (3)	
C19	0.77841 (19)	0.05550 (15)	0.43550 (12)	0.0210 (3)	
C20	0.6136 (2)	−0.14884 (16)	0.41616 (14)	0.0314 (4)	
H20A	0.5696	−0.2026	0.4612	0.038*	
H20B	0.6949	−0.1918	0.3703	0.038*	
H20C	0.5154	−0.1354	0.3702	0.038*	

### Atomic displacement parameters (Å^2^)

**Table:**

	*U*^11^	*U*^22^	*U*^33^	*U*^12^	*U*^13^	*U*^23^
S1	0.02110 (18)	0.01460 (17)	0.01551 (17)	0.00158 (13)	0.00185 (13)	0.00366 (13)
O1	0.0381 (7)	0.0269 (6)	0.0176 (5)	0.0025 (5)	−0.0021 (5)	0.0001 (5)
O2	0.0315 (6)	0.0189 (5)	0.0189 (5)	0.0002 (4)	−0.0004 (4)	0.0010 (4)
O3	0.0232 (5)	0.0212 (5)	0.0227 (5)	0.0017 (4)	0.0056 (4)	0.0091 (4)
O4	0.0415 (7)	0.0223 (6)	0.0243 (6)	0.0063 (5)	0.0025 (5)	−0.0023 (5)
O5	0.0300 (6)	0.0194 (5)	0.0242 (5)	0.0078 (4)	0.0048 (4)	0.0052 (4)
N1	0.0187 (6)	0.0191 (6)	0.0189 (6)	0.0035 (5)	−0.0025 (5)	0.0009 (5)
C1	0.0182 (7)	0.0201 (7)	0.0203 (7)	0.0006 (5)	0.0029 (5)	0.0087 (6)
C2	0.0268 (8)	0.0227 (8)	0.0274 (8)	0.0053 (6)	0.0072 (6)	0.0100 (6)
C3	0.0292 (8)	0.0280 (8)	0.0400 (9)	0.0088 (7)	0.0099 (7)	0.0196 (7)
C4	0.0251 (8)	0.0404 (10)	0.0338 (9)	0.0062 (7)	0.0037 (7)	0.0243 (8)
C5	0.0214 (7)	0.0342 (9)	0.0213 (7)	0.0020 (6)	0.0018 (6)	0.0119 (7)
C6	0.0153 (6)	0.0233 (7)	0.0200 (7)	−0.0004 (5)	0.0019 (5)	0.0081 (6)
C7	0.0200 (7)	0.0223 (7)	0.0169 (7)	−0.0005 (6)	0.0028 (5)	0.0026 (6)
C8	0.0200 (7)	0.0172 (7)	0.0179 (7)	0.0014 (5)	0.0017 (5)	0.0006 (5)
C9	0.0225 (7)	0.0201 (7)	0.0222 (7)	0.0022 (6)	0.0058 (6)	0.0037 (6)
C10	0.0453 (10)	0.0215 (8)	0.0359 (9)	0.0137 (7)	0.0117 (8)	0.0105 (7)
S2	0.02101 (18)	0.01819 (18)	0.01673 (17)	0.00130 (13)	0.00248 (13)	0.00623 (13)
O6	0.0270 (6)	0.0265 (6)	0.0175 (5)	0.0004 (5)	0.0052 (4)	0.0045 (4)
O7	0.0216 (5)	0.0304 (6)	0.0277 (6)	0.0065 (4)	0.0061 (4)	0.0128 (5)
O8	0.0328 (6)	0.0254 (6)	0.0168 (5)	0.0017 (5)	0.0022 (4)	0.0069 (4)
O9	0.0244 (5)	0.0298 (6)	0.0193 (5)	−0.0009 (5)	0.0018 (4)	0.0019 (4)
O10	0.0362 (6)	0.0198 (5)	0.0211 (5)	−0.0044 (5)	−0.0014 (5)	0.0044 (4)
N2	0.0358 (7)	0.0173 (6)	0.0172 (6)	−0.0004 (5)	0.0012 (5)	0.0065 (5)
C11	0.0184 (7)	0.0171 (7)	0.0224 (7)	0.0042 (5)	0.0023 (5)	0.0080 (6)
C12	0.0247 (7)	0.0204 (7)	0.0236 (7)	0.0028 (6)	0.0036 (6)	0.0032 (6)
C13	0.0240 (8)	0.0193 (7)	0.0326 (8)	−0.0013 (6)	0.0021 (6)	0.0024 (6)
C14	0.0209 (7)	0.0236 (8)	0.0358 (9)	0.0023 (6)	0.0085 (6)	0.0102 (7)
C15	0.0221 (7)	0.0222 (7)	0.0252 (7)	0.0054 (6)	0.0066 (6)	0.0077 (6)
C16	0.0185 (7)	0.0175 (7)	0.0217 (7)	0.0053 (5)	0.0019 (5)	0.0068 (6)
C17	0.0184 (7)	0.0211 (7)	0.0176 (7)	0.0055 (5)	0.0020 (5)	0.0051 (6)
C18	0.0228 (7)	0.0191 (7)	0.0174 (7)	0.0030 (6)	0.0004 (5)	0.0039 (6)
C19	0.0188 (7)	0.0219 (7)	0.0214 (7)	0.0039 (6)	0.0008 (5)	0.0048 (6)
C20	0.0410 (10)	0.0193 (8)	0.0267 (8)	−0.0059 (7)	−0.0052 (7)	0.0026 (6)

### Geometric parameters (Å, °)

**Table:**

S1—O2	1.4318 (12)	S2—O7	1.4310 (12)
S1—O3	1.4386 (11)	S2—O8	1.4356 (12)
S1—N1	1.6139 (14)	S2—N2	1.6170 (15)
S1—C1	1.7581 (15)	S2—C11	1.7521 (15)
O1—C7	1.3462 (18)	O6—C17	1.3426 (18)
O1—H1O	0.81 (2)	O6—H6O	0.81 (2)
O4—C9	1.2273 (19)	O9—C19	1.2292 (19)
O5—C9	1.3246 (19)	O10—C19	1.3267 (19)
O5—C10	1.4513 (19)	O10—C20	1.4516 (19)
N1—C8	1.4192 (19)	N2—C18	1.4216 (19)
N1—H1N	0.81 (2)	N2—H2N	0.83 (2)
C1—C2	1.389 (2)	C11—C12	1.388 (2)
C1—C6	1.404 (2)	C11—C16	1.407 (2)
C2—C3	1.389 (2)	C12—C13	1.392 (2)
C2—H2	0.9500	C12—H12	0.9500
C3—C4	1.387 (3)	C13—C14	1.391 (2)
C3—H3	0.9500	C13—H13	0.9500
C4—C5	1.382 (3)	C14—C15	1.382 (2)
C4—H4	0.9500	C14—H14	0.9500
C5—C6	1.403 (2)	C15—C16	1.397 (2)
C5—H5	0.9500	C15—H15	0.9500
C6—C7	1.464 (2)	C16—C17	1.466 (2)
C7—C8	1.363 (2)	C17—C18	1.363 (2)
C8—C9	1.463 (2)	C18—C19	1.459 (2)
C10—H10A	0.9800	C20—H20A	0.9800
C10—H10B	0.9800	C20—H20B	0.9800
C10—H10C	0.9800	C20—H20C	0.9800
			
O2—S1—O3	119.12 (7)	O7—S2—O8	118.19 (7)
O2—S1—N1	107.80 (7)	O7—S2—N2	110.26 (8)
O3—S1—N1	108.47 (7)	O8—S2—N2	108.16 (7)
O2—S1—C1	111.19 (7)	O7—S2—C11	107.46 (7)
O3—S1—C1	106.82 (7)	O8—S2—C11	110.99 (7)
N1—S1—C1	102.09 (7)	N2—S2—C11	100.30 (7)
C7—O1—H1O	105.2 (16)	C17—O6—H6O	107.2 (14)
C9—O5—C10	116.20 (13)	C19—O10—C20	116.26 (13)
C8—N1—S1	118.66 (10)	C18—N2—S2	118.32 (11)
C8—N1—H1N	120.1 (14)	C18—N2—H2N	122.7 (14)
S1—N1—H1N	117.5 (14)	S2—N2—H2N	118.5 (14)
C2—C1—C6	122.02 (14)	C12—C11—C16	121.77 (14)
C2—C1—S1	120.29 (12)	C12—C11—S2	120.58 (12)
C6—C1—S1	117.53 (11)	C16—C11—S2	117.57 (11)
C1—C2—C3	118.62 (15)	C11—C12—C13	118.93 (15)
C1—C2—H2	120.7	C11—C12—H12	120.5
C3—C2—H2	120.7	C13—C12—H12	120.5
C4—C3—C2	120.26 (16)	C14—C13—C12	119.95 (15)
C4—C3—H3	119.9	C14—C13—H13	120.0
C2—C3—H3	119.9	C12—C13—H13	120.0
C5—C4—C3	121.02 (15)	C15—C14—C13	120.86 (15)
C5—C4—H4	119.5	C15—C14—H14	119.6
C3—C4—H4	119.5	C13—C14—H14	119.6
C4—C5—C6	120.04 (15)	C14—C15—C16	120.39 (15)
C4—C5—H5	120.0	C14—C15—H15	119.8
C6—C5—H5	120.0	C16—C15—H15	119.8
C5—C6—C1	117.94 (14)	C15—C16—C11	118.04 (14)
C5—C6—C7	120.79 (14)	C15—C16—C17	121.18 (14)
C1—C6—C7	121.28 (13)	C11—C16—C17	120.75 (13)
O1—C7—C8	122.72 (14)	O6—C17—C18	123.41 (14)
O1—C7—C6	114.65 (13)	O6—C17—C16	114.53 (13)
C8—C7—C6	122.63 (13)	C18—C17—C16	122.04 (13)
C7—C8—N1	120.81 (13)	C17—C18—N2	120.21 (13)
C7—C8—C9	120.74 (14)	C17—C18—C19	121.04 (14)
N1—C8—C9	118.38 (13)	N2—C18—C19	118.75 (13)
O4—C9—O5	124.32 (14)	O9—C19—O10	123.81 (14)
O4—C9—C8	122.83 (14)	O9—C19—C18	123.27 (14)
O5—C9—C8	112.84 (13)	O10—C19—C18	112.90 (13)
O5—C10—H10A	109.5	O10—C20—H20A	109.5
O5—C10—H10B	109.5	O10—C20—H20B	109.5
H10A—C10—H10B	109.5	H20A—C20—H20B	109.5
O5—C10—H10C	109.5	O10—C20—H20C	109.5
H10A—C10—H10C	109.5	H20A—C20—H20C	109.5
H10B—C10—H10C	109.5	H20B—C20—H20C	109.5
			
O2—S1—N1—C8	−163.59 (11)	O7—S2—N2—C18	62.59 (13)
O3—S1—N1—C8	66.16 (13)	O8—S2—N2—C18	−166.78 (11)
C1—S1—N1—C8	−46.39 (13)	C11—S2—N2—C18	−50.50 (13)
O2—S1—C1—C2	−36.33 (14)	O7—S2—C11—C12	98.46 (13)
O3—S1—C1—C2	95.16 (13)	O8—S2—C11—C12	−32.17 (15)
N1—S1—C1—C2	−151.06 (12)	N2—S2—C11—C12	−146.32 (13)
O2—S1—C1—C6	148.12 (11)	O7—S2—C11—C16	−78.25 (13)
O3—S1—C1—C6	−80.39 (12)	O8—S2—C11—C16	151.13 (11)
N1—S1—C1—C6	33.39 (13)	N2—S2—C11—C16	36.98 (13)
C6—C1—C2—C3	2.7 (2)	C16—C11—C12—C13	1.9 (2)
S1—C1—C2—C3	−172.61 (12)	S2—C11—C12—C13	−174.65 (12)
C1—C2—C3—C4	0.1 (2)	C11—C12—C13—C14	0.3 (2)
C2—C3—C4—C5	−1.9 (3)	C12—C13—C14—C15	−1.7 (2)
C3—C4—C5—C6	1.0 (2)	C13—C14—C15—C16	0.9 (2)
C4—C5—C6—C1	1.7 (2)	C14—C15—C16—C11	1.2 (2)
C4—C5—C6—C7	−178.64 (14)	C14—C15—C16—C17	−176.71 (14)
C2—C1—C6—C5	−3.6 (2)	C12—C11—C16—C15	−2.7 (2)
S1—C1—C6—C5	171.83 (11)	S2—C11—C16—C15	174.00 (11)
C2—C1—C6—C7	176.74 (14)	C12—C11—C16—C17	175.28 (14)
S1—C1—C6—C7	−7.80 (18)	S2—C11—C16—C17	−8.05 (18)
C5—C6—C7—O1	−12.3 (2)	C15—C16—C17—O6	−17.4 (2)
C1—C6—C7—O1	167.33 (13)	C11—C16—C17—O6	164.76 (13)
C5—C6—C7—C8	167.84 (14)	C15—C16—C17—C18	161.26 (14)
C1—C6—C7—C8	−12.5 (2)	C11—C16—C17—C18	−16.6 (2)
O1—C7—C8—N1	−179.89 (14)	O6—C17—C18—N2	−177.69 (13)
C6—C7—C8—N1	0.0 (2)	C16—C17—C18—N2	3.8 (2)
O1—C7—C8—C9	−3.0 (2)	O6—C17—C18—C19	2.6 (2)
C6—C7—C8—C9	176.87 (13)	C16—C17—C18—C19	−175.92 (13)
S1—N1—C8—C7	34.08 (19)	S2—N2—C18—C17	34.94 (19)
S1—N1—C8—C9	−142.91 (12)	S2—N2—C18—C19	−145.32 (12)
C10—O5—C9—O4	−3.7 (2)	C20—O10—C19—O9	2.1 (2)
C10—O5—C9—C8	176.75 (13)	C20—O10—C19—C18	−176.20 (13)
C7—C8—C9—O4	3.3 (2)	C17—C18—C19—O9	−5.4 (2)
N1—C8—C9—O4	−179.71 (14)	N2—C18—C19—O9	174.86 (14)
C7—C8—C9—O5	−177.16 (13)	C17—C18—C19—O10	172.87 (14)
N1—C8—C9—O5	−0.17 (19)	N2—C18—C19—O10	−6.9 (2)

### Hydrogen-bond geometry (Å, °)

**Table:**

*D*—H···*A*	*D*—H	H···*A*	*D*···*A*	*D*—H···*A*
O1—H1O···O4	0.81 (2)	1.86 (2)	2.600 (2)	152 (2)
N1—H1N···O9	0.81 (2)	2.22 (2)	2.994 (2)	162 (2)
O6—H6O···O9	0.81 (2)	1.91 (2)	2.634 (2)	147 (2)
N2—H2N···O3^i^	0.83 (2)	2.13 (2)	2.966 (2)	175 (2)
C4—H4···O8^ii^	0.95	2.36	3.259 (2)	158
C20—H20A···O2^i^	0.98	2.51	3.267 (2)	134

Symmetry codes: (i) −*x*+1, −*y*, −*z*+1; (ii) *x*−1, *y*, *z*−1.
